# Effectiveness of ZnPc and of an amine derivative to inactivate Glioblastoma cells by Photodynamic Therapy: an *in vitro* comparative study

**DOI:** 10.1038/s41598-019-39390-0

**Published:** 2019-02-28

**Authors:** Fabiola N. Velazquez, Mariana Miretti, Maria T. Baumgartner, Beatriz L. Caputto, Tomas C. Tempesti, César G. Prucca

**Affiliations:** 1 CIQUIBIC (CONICET), Departamento de Química Biológica Ranwel Caputto, Facultad de Ciencias Químicas, Universidad Nacional de Córdoba, Córdoba, Argentina; 2 INFIQC (CONICET), Departamento de Química Orgánica, Facultad de Ciencias Químicas, Universidad Nacional de Córdoba, Córdoba, Argentina

## Abstract

Glioblastoma multiforme is considered to be one of the most aggressive types of tumors of the central nervous system, with a poor prognosis and short survival periods of ~ one year. The current protocol for glioblastoma treatment includes the surgical excision of the primary tumor followed by radio and chemotherapy. Photodynamic therapy (PDT) is considered a promising strategy for the treatment of several types of tumors. Phthalocyanines (Pcs) are good photosensitizers (PSs) for PDT because they induce cell death in several cellular models. ZnPc (Zn(II)phthalocyanine) is a well-known Pc, extensively tested in different cells and tumor models, but its evaluation on a glioblastoma model has been poorly studied. Herein, we compare the capacity of ZnPc and one of its derivatives, Zn(II)tetraminephthalocyanine (TAZnPc), to photoinactivate glioblastoma cells (T98G, MO59, LN229 and U87-MG) in culture. We measured the cellular uptake, the toxicity in the dark and the subcellular localization of the different Pcs, as well as the clonogenic capacity of surviving cells after PDT. The mechanism of cell death induced after PDT was determined by measuring caspase 3 activation, DNA fragmentation, phosphatidylserine externalization, mitochondrial morphological changes and loss of mitochondrial membrane potential as well as lysosomal membrane integrity. Overall, ZnPc and TAZnPc present good properties to be used as PSs with photoinactivation capacity on glioblastoma cells.

## Introduction

Gliomas account for approximately 70% of the new cases of primary brain tumors diagnosed in adults in the United States each year^1^. Glioblastomas multiforme (classified by the World Health Organization as type IV glioma) are one of the most common and aggressive forms of tumors of the central nervous system and, in the United States, more than 10,000 new cases are reported every year^2^. The location of these tumors in critical areas of the brain makes them difficult to be removed by surgery whereas the blood-brain barrier limits the access of drugs to reach their site of action thus complicating even more the possibility of controlling their growth^3,4^.

At present, the protocol for treatment of Glioblastomas multiforme involves surgical resection followed by chemo and radiotherapy that results in an average survival time of approximately 14.6 months^5^. Due to the highly invasive nature of these tumors, the surgical elimination of the primary tumor bulk is usually not curative and the presence of invasive infiltrating cells leads to the development of secondary tumors either close or distant to the location of the primary one. In addition, as with other tumors, cancer stem cells (CSCs) play a role in the growth, maintenance and metastasis of these tumors, as well as in the resistance to radio and chemotherapy and tumor recurrence after treatment^6–8^.

Photodynamic therapy (PDT) is an effective strategy for the treatment of several cancers, microbial diseases, diagnosis, as well as for cosmetic purposes^9^. PDT involves a non-toxic compound known as photosensitizer and visible light of the wavelength absorbed by the PS which in the presence of oxygen leads to the generation of singlet oxygen (^1^O_2_) and/or reactive oxygen species (ROS) that can damage cellular constituents leading to cell death^10,11^ followed by tumor regression^12–15^. As these reactions occur only in the local area of the light-absorbing photosensitizer, the biological responses are limited to the area that has been irradiated. Ideal PS should be accumulated in target tissues and rapidly eliminated to prevent secondary effects related to photosensitivity^16^.

The main purpose of using PDT to treat tumors is to trigger the destruction of tumor cells by induction of cell death. Several factors influence the type of cell death that occurs after PDT: the properties, concentration, and subcellular localization of the PS, the oxygen available at the site of irradiation, the dose of light delivered and the cell type^17^. After PDT, cells can undergo at least two types of cell death, that is, apoptosis or necrosis. The first refers to the physiological cell death that occurs without triggering inflammation or immunological responses whereas necrosis is a fast, non-regulated and aggressive form of cell death, commonly associated with inflammatory processes^18^.

Since PDT effects are limited to the site of irradiation, the use of this therapeutic approach for the treatment of high infiltrating gliomas has become a topic of interest for many researchers. Several studies have been performed showing the potentiality of the therapy using different PSs^19–24^. Phthalocyanines (Pcs) and their derivatives have been considered excellent PSs (second generation) for PDT in numerous types of tumors. This type of molecule strongly absorbs in the red and near infrared regions of the visible spectrum, which corresponds to the tissue optical window^12,25,26^. In addition, Pcs present high photo and chemical stability^27,28^. Zn(II)phthalocyanine (ZnPc) is a well-known Pc and several reports have proved its properties as PS for PDT^13,28,29^. However, to the best of our knowledge, only a few reports analyzed the effectiveness of Pcs on a glioblastoma cell model^30^.

The aim of the present study was to evaluate the efficacy of two phthalocyanines: ZnPc and Zn(II)tetraminephthalocyanine (TAZnPc) to photo-inactivate glioblastoma cells *in vitro*. Dark toxicity, cellular uptake and subcellular localization of ZnPc and TAZnPc were determined. In addition, effectiveness of these Pcs to reduce cell viability in culture as well as the clonogenic capacity of post-PDT surviving cells were examined. Finally, the type of cell death triggered after PDT was assessed evaluating the disruption of mitochondrial integrity, loss of mitochondrial membrane potential (MMP), activation of caspase 3, loss of lysosomal membrane potential and DNA fragmentation.

## Results

**Figure 1:**
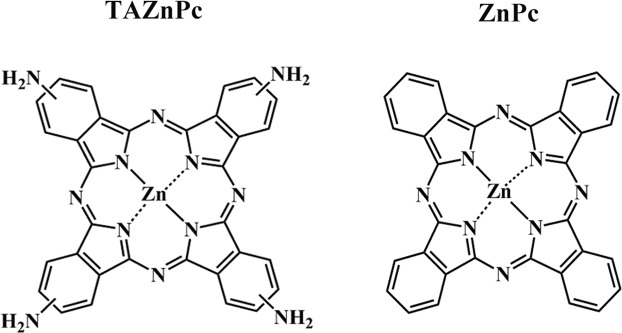
Phthalocyanines. Molecular structures of the two phthalocyanines used in this study, TAZnPc (left) and ZnPc (right).

The Pcs (Fig. 1) used in this work were synthetized as described under Materials and Methods. The absorption spectra of TAZnPc in DMF presents a higher red-shifted λ_max_ (702 nm) compared to ZnPc (670 nm)^31^ (Supplemental Figs 1 and 2). Fluorescence quantum yields (Ф_F_) of TAZnPc was calculated Ф_F(TAZnPc)_ = 0.03, by comparative method using ZnPc as standard (Ф_F(ZnPc)_ = 0.17)^32^. The singlet oxygen production in DMF was determined by TAZnPc, Φ_Δ_ = 0.45 ± 0.02 (Supplemental Fig. 3), this value is similar to that of non-modified ZnPc (0.56)^33^.

### Dark cytotoxicity of photosensitizers

**Figure 2:**
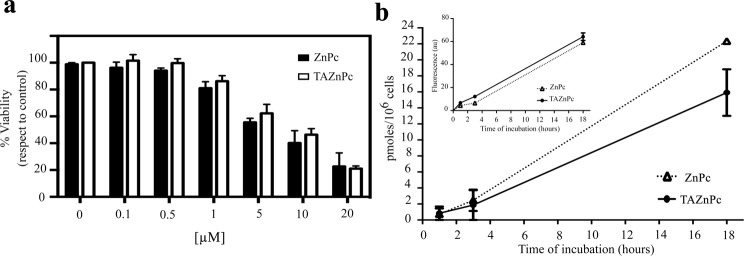
(**a**) Dark toxicity of photosensitizers. T98G cells were incubated in the dark during 18 hours with different concentrations of TAZnPc (white bars) or ZnPc (black bars) dissolved in DMEM supplemented with 4% of FBS plus antibiotics. Then the cells were washed and viability assessed using alamarBlue as described under Materials and Methods. Results are presented as mean percentage of viability with respect to the control (cells without Pc) ± SEM of three independent experiments performed in triplicate. (**b**) Cellular uptake was measured by two methods as described under Materials and Methods. Values are presented as pmoles/10^6^ cells ± SEM of a representative experiment. Flow cytometer results are presented in the inset graph. The results are the mean ± SD of the fluorescence intensity change compared to the control of a representative experiment out of two done in triplicate.

To evaluate if the Pcs used in this study affect cell viability in the absence of irradiation, we examined the effect of the addition of Pcs to glioma cell cultures. Cells were incubated during 18 hours with different concentrations of the Pc (in the range of 0–20 µM) diluted in Dulbecco’s modified Eagle medium (DMEM) supplemented with 4% FBS and cell viability measured. Results in Fig. 2a show that, for all Pcs evaluated in T98G cells, concentrations of 0.5 µM was not cytotoxic in the absence of irradiation, with a survival fraction higher than 90% as compared to control cells cultured in the absence of Pc. However, higher concentrations of photosensitizer lead to a reduction in cell viability, showing survival fractions lower than 90%. Based on these results, hereafter, all the following experiments were performed using Pc concentrations ≤ 0.5 µM. Results obtained in T98G cells are similar to those observed in three additional glioma cell lines (Supplementary Fig. 5).

### Cellular uptake

To evaluate the cellular uptake of the different Pcs, T98G cells were incubated during different times in the presence of each Pc (0.5 μM) and the relative amount of each Pc incorporated into the cells was evaluated both by the direct measurement of fluorescence and also by flow cytometry. As can be observed in Fig. 2b, the results obtained by both methodologies indicate that ZnPc and TAZnPc uptake increased over time.

### Subcellular localization

**Figure 3:**
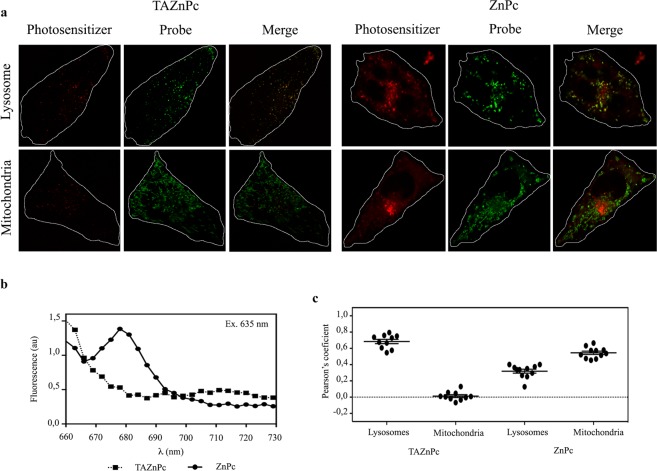
Subcellular localization of TAZnPc and ZnPc in T98G cells (**a**) Subcellular localization of Pcs. Cells were incubated with Pcs at 0.5 μM for 18 hours, then, where incubated with organelles specific probes (Lysosome upper row and Mitochondria lower row) and observed *in vivo* under a confocal microscope. Pcs fluorescence (column 1 and 4), organelle-specific probe fluorescence (column 2 and 5), and merged images (column 3 and 6) are presented. (**b**) Fluorescence intensity profile. In order to confirm the identity of the observed intracellular fluorescence, we excited with a laser at 635 nm and recorded the fluorescence at different wavelength. (**c**) Pearson’s coefficient between Pc signal and the organelle-specific probe signal was calculated using Image J.

The subcellular accumulation and localization of the sensitizers is critical to evaluate the potentiality for PDT, since the singlet oxygen generated after illumination presents a short lifetime and a migration distance of approximately 1 μm^34^. Consequently, the subcellular localization of TAZnPc and ZnPc was examined by confocal microscopy using organelle-specific probes. As can be observed in Fig. 3, TAZnPc and ZnPc are accumulated both in cytoplasm and in the perinuclear region. No Pcs was detected in the nucleus. When we analyzed the possible co-localization of the PS signal with the organelle-specific probes we observed that TAZnPc accumulates preferentially in lysosomes (Pearson’s coefficient 0.7539 ± 0.012), whereas ZnPc accumulates preferentially in mitochondria (Pearson’s coefficient 0.7336 ± 0.017) but also partially in lysosomes (Pearson’s coefficient 0.4953 ± 0.011) (Fig. 3a,c). Moreover, we analyzed the fluorescence spectra in order to assess the identity of the fluorescence. As can be seen in the fluorescence spectra presented in the middle panel, when cells were excited with a laser at 635 nm, cells incubated with ZnPc present an intracellular signal with a maximum at 675 nm whereas the cells incubated with TAZnPc show a maximum at 711 nm, confirming the Pc specific fluorescence (Fig. 3b).

### Photocytotoxicity

**Figure 4:**
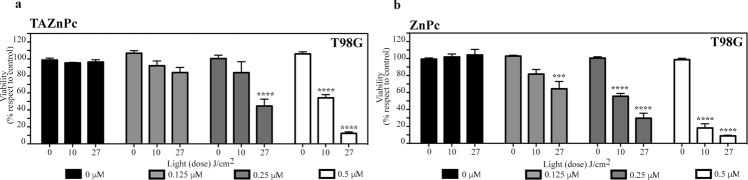
Effect of the photosensitizers on T98G cell viability. T98G cells were incubated in the presence of 0.125 (light gray), 0.25 (dark gray) or 0.5 µM (white) of: (**a**) TAZnPc and (**b**) ZnPc dissolved in DMEM supplemented with 4% FBS plus antibiotics during 18 hours. Then the medium was replaced with fresh medium without Pcs and the cells irradiated using two different light doses: 10 or 27 J/cm^2^. Cell viability was measured 24 hours after PDT using alamarBlue as described under Materials and Methods. Results are the mean ± SEM of cell viability with respect to the control (non-irradiated cells in absence of Pc) of three independent experiments performed in triplicate. ***p < 0.005 and ****p < 0.001 with respect to the control (non irradiated cells in the absence of Pc) using two-way ANOVA with Dunett’s post-test.

The photocytotoxicity of the different Pcs in T98G cells was assessed at three different Pc concentrations 0.125, 0.25 and 0.5 µM. After 18 hours of incubation with one of the Pcs, cells were irradiated using two light doses: 10 or 27 J/cm^2^ and viability measured 24 hours after irradiation. Results show a clear cytotoxic effect on cells subjected to the combination of light and either TAZnPc or ZnPc (Fig. 4a,b, respectively). Both Pcs were innocuous in the absence of light (0 J/cm^2^) at the Pc concentrations tested, with survival fraction higher that 90%. In addition, neither light doses don’t reduce cell viability in absence of Pc indicating that there was no detectable thermal effect during the irradiation of the cells. As can be observed, a ~90% reduction in cell survival is achieved using either ZnPc or TAZnPc at 0.5 µM combined with a light dose of 27 J/cm^2^, meanwhile, when a lower light dose was used (10 J/cm^2^) ZnPc-PDT was able to reduce the cell viability by ~80% whereas TAZnPc was less effective to photoinactivate T98G cells using a lower light dose (10 J/cm^2^) showing a reduction of ~46% in cell viability. These results shown a clear relation between the Pc concentration and the light dose delivered in the photo inactivation capacity.

### Clonogenic Assay

**Figure 5:**
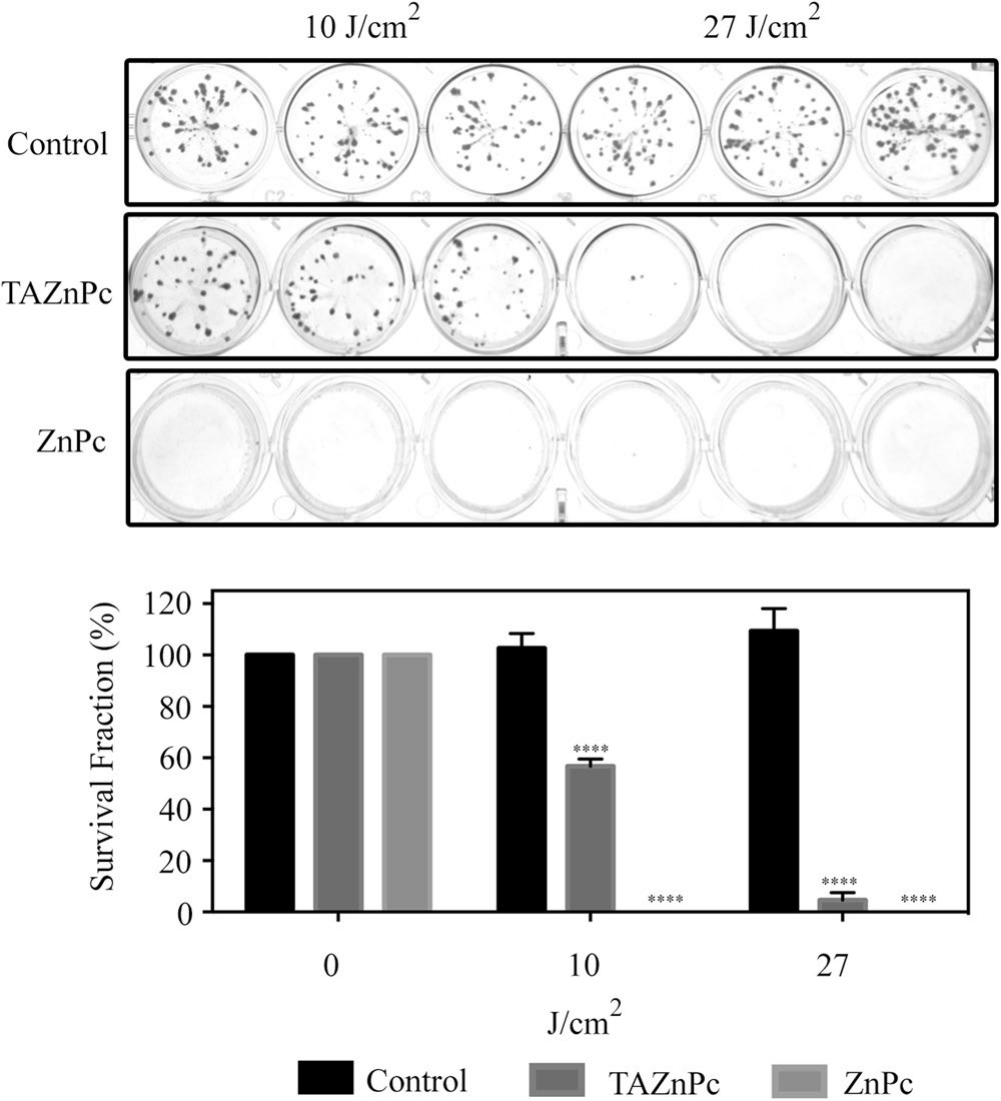
Clonogenic Assay. T98G cells were treated with ZnPc or TAZnPc 0.5 µM, irradiated using 10 or 27 J/cm^2^ and plated 24 hours post-PDT. Top panel shows representative images of wells in which 50 cells were seeded. Lower panel shows quantification of cell survival. Results are presented as the mean fraction of cells that survived ± SEM of one of two independent experiments performed in triplicate. ****p < 0.0001 with respect to the control (irradiated cells in the absence of Pc) using two-way ANOVA with Dunnett’s post-test.

Since the recurrence of tumors after treatment is based on the capacity of surviving cells to proliferate, we evaluated the clonogenic capacity of T98G cells after PDT. For this, cells were treated with Pcs at 0.5 µM, irradiated using two light doses (10 and 27 J/cm^2^), and 24 hours after PDT, cells were washed, counted and plated. 8–10 days later, colony formation was assessed and the survival fraction determined for each treatment. As can be seen in Fig. 5, no colonies were observed in cells treated with ZnPc combined with both light doses. However, as was observed with the alamarBlue assay, the combination of TAZnPc with 10 J/cm^2^ reduces cell viability in ~45%, whereas the application of a higher light dose completely abolishes the colony formation capacity of T98G cells.

### Mode of cell death triggered by PDT

Since it has been previously reported that PDT treatment of tumor cells either *in vitro* or *in vivo* can induce cell death by different mechanisms^18^, we evaluated the pathway of cell death triggered upon treatment of cells with the combination of Pc plus light.

**Figure 6:**
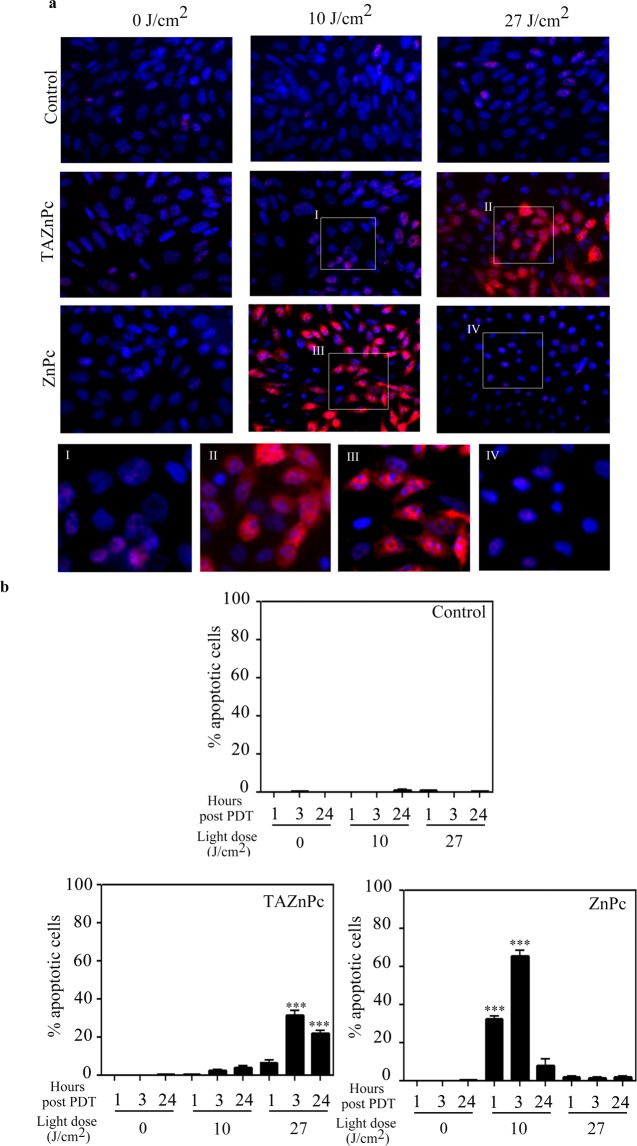
Apoptosis induction: nuclei fragmentation and caspase 3 activation. T98G cells were incubated with TAZnPc or ZnPc dissolved in DMEM supplemented with 4% FBS plus antibiotics at a concentration of 0.5 µM during 18 hours. Then the medium was replaced and the cells irradiated using two light doses: 10 J/cm^2^ or 27 J/cm^2^. Cells were fixed at different times post PDT and activation of caspase 3 and nuclei fragmentation were monitored using anti-CC3 antibody and DAPI staining respectively, as described under Materials and Methods. (**a**) Representative photomicrographs of T98G cells without Pc (first row), with TAZnPc (second row) or with ZnPc (third row), non-irradiated (first column) or irradiated using 10 J/cm^2^ (second column) or 27 J/cm^2^ (third column) stained using anti-CC3 antibody (red) and DAPI (blue). Magnifications of boxed areas are shown (I-IV, fourth row). (**b**) Quantification of apoptotic cells (CC3+ cells with fragmented nuclei) represented as mean % ± SD of apoptotic nuclei over the total number of nuclei (DAPI positive). ***p < 0.001 with respect to the control using two way ANOVA with Tukey’s post-test.

To determine if apoptosis was induced in T98G cells using the different Pcs for PDT, treated cells were fixed at different times post irradiation (1, 3 or 24 hours) and stained using anti cleaved caspase 3 (CC3) antibody and 4′,6-diamidino-2-phenylindole (DAPI). Fragmented nuclei positive for CC3 were considered as apoptotic cells. As can be seen in Fig. 6a, PDT using either ZnPc or TAZnPc induces programmed cell death in glioblastoma cell cultures. The highest percentage of adherent cells undergoing apoptosis was observed 3 hours after irradiation with 10 J/cm^2^ in cells treated with ZnPc (~65%). However, samples measured 24 hours post PDT using the same light dose showed less CC3+ fragmented nuclei cells since those that had previously undergone apoptosis become detached and were not being detected at this time point by the assay. Small amounts (<5%) of attached CC3+ cells with fragmented nuclei were observed in samples treated with ZnPc and 27 J/cm^2^. Cells treated with TAZnPc showed the highest amount of CC3+ fragmented nuclei 3 hours after irradiation with 27 J/cm^2^ (~31%).

**Figure 7:**
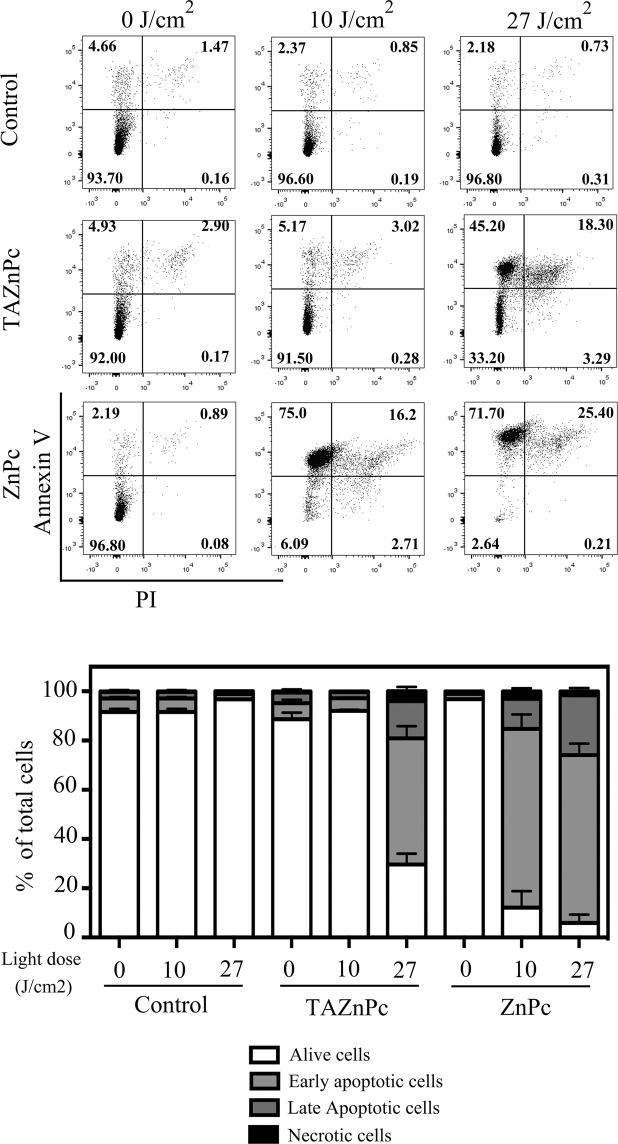
Apoptosis induction: Annexin V/PI staining. T98G cells were incubated with TAZnPc or ZnPc dissolved in DMEM supplemented with 4% FBS plus antibiotics at a concentration of 0.5 µM during 18 hours. Then, medium was replaced by fresh medium not containing Pcs and cells were irradiated using two light doses: 10 J/cm^2^ or 27 J/cm^2^. Three hours after irradiation, floating and attached cells were collected and washed. Then cells were stained using Annexin V conjugated to FITC (staining used to determine exposed phosphatidylserine) and PI and analyzed by flow cytometry. A representative dot plot graph in which cells in the lower left quadrant are considered living cells (Annexin V−PI−), cells in the upper left quadrant are early apoptotic cells (Annexin V+PI−), cells in the upper right quadrant are late apoptotic cells (Annexin V+PI+) and cells in the lower right quadrant are necrotic cells (Annexin V− PI+). The mean percentage ± SEM of cells in each quadrant with respect to the total number of cells is represented below in the bar graph. The result is a representative experiment of three performed in triplicate. Individual plots are presented in Supplementary Fig. 6.

Since only attached cells were taken into account in the above-mentioned analysis, we additionally measured early signals (three hours after PDT) of apoptosis by detection of phosphatidylserine externalization (Annexin V) and propidium iodide (PI) staining followed by flow cytometry analysis. As shown in Fig. 7, phosphatidylserine externalization was observed after irradiation with 10 or 27 J/cm^2^ in cells treated with ZnPc. Upon the application of a light dose of 10 J/cm^2^, 72.6 ± 5.8% of cells classified as early apoptotic cells, whereas 12.83 ± 2.48% of de total cell population showed signs of late apoptosis. On the other hand, when a higher light dose was used, we observed 68.2 ± 4.6 and 24.3 ± 0.6% of early and late apoptotic cells respectively. However, in the case of TAZnPc, a significant population of Annexin V positive cells was observed only after irradiation with 27 J/cm^2^. This population was composed by 51.2 ± 4.9 and 15.1 ± 2.4% of early and late apoptosis cells respectively. These results are in line with the above-mentioned observations regarding the activation of caspase 3 and nuclei fragmentation indicating that higher light doses are required to induce apoptosis upon TAZnPc treatment than with ZnPc. A non-significant cell population underdoing necrosis was observed in both treatments with both Pcs, suggesting that the apoptotic pathway is the preferential cell death mechanism triggered after irradiation of the cells.

### Mitochondrial disruption after PDT

**Figure 8:**
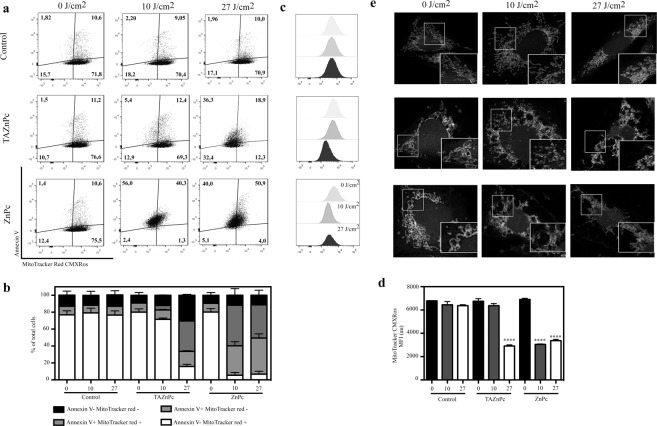
Apoptosis induction: Annexin V/MitoTracker red CMXRos staining: (**a**) Cells were treated as described in Fig. 7. Three hours after PDT, cells were collected, washed and incubated during 30 min with MitoTracker red CMXRos dissolved in DMEM supplemented with 10% FBS plus antibiotics. Then, cells were washed, stained using Annexin V conjugated to FITC and analyzed by flow cytometry. The results of a representative experiment are represented in dot plot graphs of Annexin V vs MitoTracker red CMXRos. Cells undergoing apoptosis start to exteriorize phosphatidylserine and become Annexin V positive (upper right and left panel). The loss of mitochondrial membrane potential (MMP) can be evidenced by the decrease in MitoTracker red CMXRos staining. (**b**) The mean percentage ± SEM of cells in each quadrant with respect to the total number of cells is represented in the bar graph. (**c**) Representative fluorescence histograms of total cells stained with MitoTracker red CMXRos. Note that TAZnPc (second row) and ZnPc (third row) plus light induce a shift in the histogram to lower values suggesting a loss of MMP. (**d**) The mean fluorescence intensity MFI ± SEM of Mitotracker Red CMXRos is represented in the bar graph. ****p < 0.001 with respect to non-irradiated control cells using one way ANOVA with Dunnett´s post-test (**e**) Photomicrographs of T98G cells treated with TAZnPc (second row) or ZnPc (third row) stained with MitoTracker red CMXRos to show mitochondrial morphology three hours after irradiation with 0 J/cm^2^ (left), 10 J/cm^2^ (middle) or 27 J/cm^2^ (right). Magnifications of boxed areas are shown in the lower right corner of each photomicrograph.

It has been previously proposed that the cell damage triggered by PDT begins at the subcellular compartment in which the Pc is accumulated^35^. Since previous reports suggest that ZnPc could be localized in mitochondria^36^, Golgi^37^, lysosomes and endoplasmic reticulum^38^, and considering that we observed that there is colocalization of ZnPc with a mitochondria-specific probe (Fig. 3a) we evaluated the effect of PDT on mitochondria, a key organelle involved in apoptosis. As can be observed in Fig. 8a,b, an increase in the number of cells positive for Annexin V (early apoptosis signal) is accompanied by a decrease in the number of cells positive for the mitochondrial probe MitoTracker red CMXRos in cells treated either with ZnPc or TAZnPc. Interestingly, this observation is already evident at a low light dose for ZnPc-treated cells but only at the higher doses for cells treated with TAZnPc in accordance with the results shown in Fig. 7. Histograms of fluorescence intensity for MitoTracker Red in cells treated with either of the Pcs mentioned above show a loss of signal, indicating a decrease in mitochondrial membrane potential (MMP) after PDT (Fig. 8c,d). Moreover, we assessed mitochondrial morphology by confocal microscopy and, as shown in Fig. 8e, a clear morphological change was observed in cells treated with either ZnPc or TAZnPc. Additionally, a light dose dependence on injury was observed supporting our previous results. Taken together, these results show that mitochondria are clearly affected by PDT using ZnPc and TAZnPc.

### Lysosomal disruption after PDT

**Figure 9:**
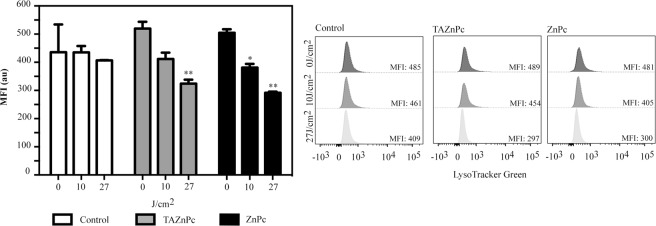
Lysosome disruption after PDT. T98G cells were incubated with TAZnPc (grey bars) or ZnPc (black bars) for 18 hours. Then, the media was discarded and cells incubated with Lysotracker for two hours to allow the accumulation of the probe in lysosomes. After the incubation, media was changed, and the cells irradiated with 10 or 27/cm^2^. One hour after irradiation total cells were collected and the Lysotracker Green fluorescence was assessed using flow cytometry. Quantification of the mean fluorescence intensity (MFI) is shown in the bar graph. Results are presented as mean ± SEM of MFI of a representative experiment made in triplicate. **p < 0.01, *p < 0.05 with respect to the cells without irradiation (0 J/cm^2^) using Two way ANOVA with Dunnett’s post test.

Lysosomes are critical organelles for the cell. These subcellular compartments plays critical roles in controlling cell homeostasis as well as in triggering cell death^39^. Since we observed lysosomal accumulation of both Pcs, we analyzed the effect of the irradiation on these organelles. For this, we used the Lysotracker green retention as an indicator of lysosome membrane integrity and membrane potential changes. Since we observed that there is a rapid induction of apoptosis after irradiation in cells incubated with ZnPc or TAZnPc, we measured the early (1 hour after PDT) loss of Lysotracker green fluorescence. Results shown in Fig. 9 indicate that after irradiation with 27 J/cm^2^ on cells incubated with ZnPc or TAZnPc, there is a significant reduction in the Lysotracker green signal, suggesting that lysosomes were disrupted after irradiation. In concordance with previous results, no significant loss of LysoTracker probe retention is observed in cells subjected to PDT with TAZnPc at the lower light dose or in cells irradiated without Pcs. This result suggests that this organelle is disrupted after PDT.

### DNA fragmentation after PDT

To evaluate the end-point hallmark of apoptosis, that is DNA laddering, cells treated with the different Pcs were used to isolate DNA and DNA laddering was determined. Results shown in Supplementary Fig. 7 showed no evident signals of DNA fragmentation in either of the two light doses used, in the absence of Pc (control). By contrast, cells treated with TAZnPc show a typical DNA fragmentation pattern 24 hours after irradiation with 27 J/cm^2^. In the case of cells treated with ZnPc, DNA fragmentation was also evident at 24 hours showing mainly fragments of low molecular length. However, when DNA was isolated at an early time point (6 hours after PDT with ZnPc) the laddering pattern was more evident. The pattern of DNA oligonucleotide fragmentation observed suggests differences in the apoptosis timing after irradiation of cells treated with ZnPc or with TAZnPc, being the induction of apoptosis faster in the first case (Supplementary Fig. 7).

### Photocytotoxicity in other glioma cells

**Figure 10:**
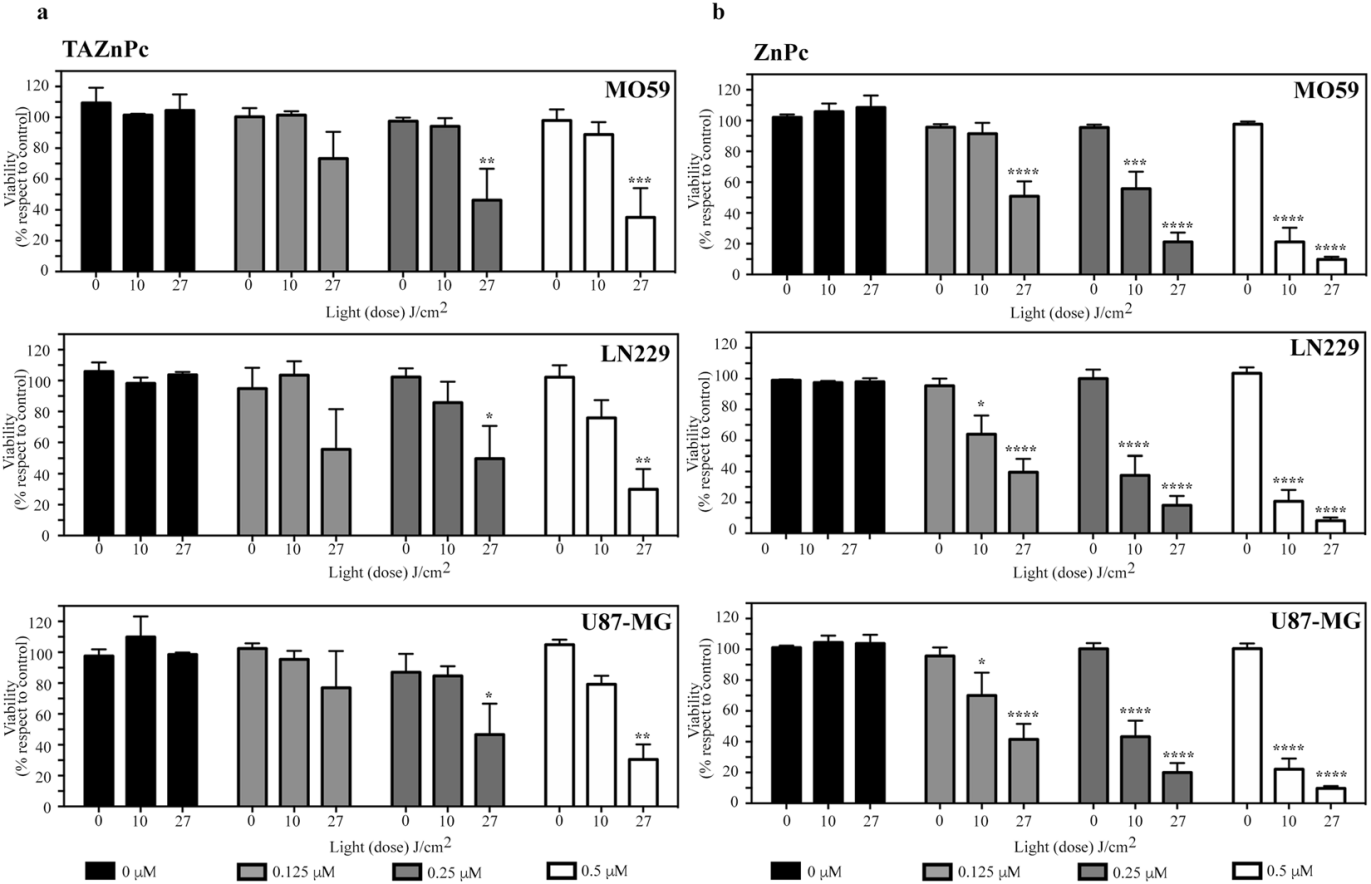
Effect of the photosensitizers on glioma cell viability. Three glioma cell lines (MO59, LN229 and U87-MG) were incubated in the presence of 0.125 (light gray), 0.25 (dark gray) or 0.5 µM (white) of: (**a**) TAZnPc or (**b**) ZnPc dissolved in DMEM supplemented with 4% FBS plus antibiotics during 18 hours. Then the medium was replaced with fresh medium without Pc and the cells irradiated using two different light doses: 10 or 27 J/cm^2^. Cell viability was measured 24 hours after PDT using alamarBlue as described under Materials and Methods. Results are the mean ± SEM of three independent experiments performed in triplicate. *p < 0.05 **p < 0.01 ***p < 0.005 and ****p < 0.001 with respect to the control (non irradiated cells in the absence of Pc) using two-way ANOVA with Dunnett’s post-test.

The photocytotoxicity of the different Pcs was evaluated in three additional glioma cell lines (MO59, LN229 and U87-MG). Both Pcs were evaluated at three different concentrations 0.125, 0.25 and 0.5 µM following the PDT protocol described above for T98G cells. Pcs dark toxicity was assessed in the three cell lines with results similar to the reported above for T98G cells (Supplementary Data). We observed that, similar to the results for T98G cells, both TAZnPc and ZnPc are able to trigger a reduction in cell viability when the cells are irradiated. (Fig. 10a,b, respectively). In the three cell lines analyzed, either light or Pcs alone were not able to reduce cell viability (survival fraction higher that 90%) whereas ZnPc at 0.5 µM combined with a light dose of 27 J/cm^2^ reduced ~90% of cell viability in all cell lines evaluated. In the case of TAZnPc, only ~70% of reduction was observed in the three cell lines when the highest Pc concentration and light dose were delivered. When the lower light dose was used (10 J/cm^2^), ZnPc at 0.5 µM gave similar results in all cell lines achieving a reduction of ~80% in cell viability. By contrast, TAZnPc was less effective to photoinactivate the cells with ~20% of reduction in cell viability in the cell lines examined. A non-significant reduction in cell viability was observed in all the cell lines using TAZnPc at 0.125 µM whereas ZnPc still was capable of reducing the cell viability in all glioma cell lines when used at the same concentration. A clear correlation between the Pc concentration and the light dose delivered was observed in the three cell lines in the photoinactivation capacity.

## Discussion

The development of new PSs is a mayor goal in the current research on PDT and much more so for tumors in which other therapies have resulted ineffective as is the case of brain tumors. One of the determinants for brain tumor recurrence is probably the growth pattern of this malignant tissue that is diffuse and in consequence, is not completely eliminated by surgical resection. Glioblastomas multiforme are one of the most aggressive forms of brain tumors and after diagnosis, are associated with a poor prognosis and with survival periods of approximately 14 months. In this work, we tested the potentiality of ZnPc and one of its derivatives, TAZnPc, to photoinactivate glioblastoma cells and analyzed the cellular mechanisms involved in the PDT response *in vitro*.

The penetration of light into living tissues depends on several parameters: intensity, polarization and coherence of the light source, tissue composition and hydration, tissue compression and the light wavelength used to activate the PS. It is known that some PSs that are excited at longer wavelengths have the potential to increase treatment depths in PDT of tumors^40^. We observed that TAZnPc shows a shift to higher λ_max_ in the absorption spectra as compared to ZnPc. This photochemical property allows the use of light of a longer wavelength, which deeply penetrates the tissue thus favoring the activation of Pc accumulated in cells located deep in the tissue^12,16,25,26^.

No changes in cell viability were observed with either Pcs when the cells where incubated with concentrations ≤0.5 µM. The cellular uptake of these Pcs (direct and indirectly measured by determining the amount of intracellular Pc and the change in cellular fluorescence, respectively) presents a maximum at 18 hours with non-significant changes at 24 hours (less than 10%). A recent report of Soriano *et al*.^41^ shows that ZnPc-DMF is incorporated into human lung adenocarcinoma cells by means of caveolin-mediated endocytosis and that the PS is activated by light^41^. Our results suggest that both ZnPc and TAZnPc are incorporated into T98G glioblastoma cells in a time dependent fashion. Since it has been proposed that the site of accumulation of the PS is the site of action after activation by light, we evaluated the subcellular accumulation of both phthalocyanines in T98G cells having found that ZnPc accumulates preferentially in mitochondria but also in lysosomes, results similar as those previously reported for other cell lines^36,37,42^. Interestingly, TAZnPc accumulates predominantly in the lysosomes suggesting that this organelle is the principal site of action of this PS.

Glioblastoma cell photoinactivation was observed in cell cultures treated with ZnPc as well as with TAZnPc. In addition, both ZnPc and TAZnPc show a direct relation between cell photoinactivation and Pc concentration plus light dose delivered. A reduction of ~90% in cell viability was achieved after irradiating cells previously incubated with 0.5 µM of either Pcs, with a higher light dose (27 J/cm^2^), meanwhile a light dose of 10 J/cm^2^ trigger a lower reduction in cells treated with TAZnPc as compared with those previously incubated with ZnPc.

It’s known that brain tumors, specially glioblastomas, present a higher recurrence after surgery since the complete elimination is difficult because tumor limits are diffuse and in consequence remaining tumor cells allows the growing of tumors after the initial treatment. In line with this observation, we evaluate the clonogenic capacity of treated cells after PDT and the results obtained indicate that cells treated with ZnPc do not retain proliferation capability after PDT using either light doses. Similar results were found in cells treated with TAZnPc and higher light-dose, however, a clonogenic capacity was observed in lower light dose-treated cells (10 J/cm^2^). All together, these results can be correlated with the singlet oxygen production observed (Φ_Δ_), which indicates that TAZnPc has a reduced capacity to produce this high-energy state molecular oxygen specie as compared with ZnPc.

Cell death by the apoptotic pathway constitutes the most desirable outcome of tumor treatment since it represents the physiological cell death mechanism and does not trigger inflammation or immune response. Since PDT using Pcs triggers apoptotic events by damaging critical organelles of the cells^43^, we studied the mechanism of cell death in T98G cell treated with ZnPc or TAZnPc. The apoptotic pathway involves the early activation of caspase 3, one of the mayor effector caspases^44^ and a clearly observable nucleus fragmentation. These apoptotic events were evident in cells incubated with ZnPc and irradiated with the either light dose as compared to cells treated with TAZnPc in which the caspase 3 activation together with nucleus fragmentation were evident only after irradiation with the highest light dose used. In line with this result, we observed another hallmark of apoptosis, the DNA internucleosomal fragmentation^45^, as early as 6 hours after cell treatment with ZnPc, while in cells treated with TAZnPc, the DNA laddering was clearly observed in samples obtained 24 hours after irradiation (Supplementary Data) suggesting differences in the timing of the cell death mechanism^46^.

Early markers of apoptotic events include the externalization of phosphatidylserine in the cellular membrane. In cells treated with either ZnPc or TAZnPc, we observed a rapid increase in the number of cells positive for Annexin V with the differences observed previously, that is, higher light doses must be delivered to cells incubated with TAZnPc to obtain this effect.

Mitochondria play a key role in activating apoptosis in cells^47^ and the permeabilization of the mitochondrial outer membrane represents the defining event that irrevocably commits a cell to die^48^. Previous reports, that examined the subcellular localization of Zinc phthalocyanines, suggest that this PS accumulates predominantly in mitochondria and lysosomes^36,38,42,49^. Based on the results obtained in this report regarding the subcellular localization of both Pcs and considering the evidence present in the literature that highlight the importance of lysosome disruption for the mitochondrial triggered apoptosis^39,50–54^, we evaluated the morphology and functionality of mitochondria before and after PDT. Mitochondrial morphology dramatically changes in cells treated with ZnPc or TAZnPc shortly after irradiation, suggesting that the singlet oxygen and/or ROS generated affects on the functionality of this organelle. In addition, a change in the mitochondrial membrane potential was clearly observed in cells treated with either Pcs. Both the change in the mitochondrial membrane potential and the externalization of phosphatidylserine on the external side of the cell membrane support that mitochondria is involved in the cell death triggered by the PDT in T98G cells treated with ZnPc or TAZnPc. Moreover, an early disruption of lysosome integrity was observed as soon as 1 hour after irradiation, suggesting that these phenomena could be related to the mitochondrial-associated cell death as reported previously^55^. The almost totally accumulation of TAZnPc into lysosomes could partially explain the differences in cell injury after irradiation since only when a higher light dose was delivered the response was similar to that observed in ZnPc treated cells irradiated with lower light dose. The lysosomal injury observed in TAZnPc irradiated with high light dose could be related to the mitochondrial damage reported in these cells after a 27 J/cm^2^ light dose was delivered. The capacity of both Pcs to photoinactivate glioma cells (T98G, MO59, LN229 and U87-MG) highlights the potentiality of these two compounds for PDT in brain tumors.

Pcs are considered excellent candidates to be used as PSs in PDT treatment of different types of tumors. Regarding brain tumors, several studies using different PSs were reported with promising results^56,57^, but more studies are necessary to probe the effectiveness of this therapeutic strategy as an adjuvant for the treatment of these tumors. The results presented herein support that both Pcs, ZnPc and TAZnPc, have excellent properties that justify testing them in an animal model of glioblastoma. Furthermore, the shift in the λ_max_ absorption observed for TAZnPc is a promising feature for the activation of the Pc accumulated in deeper portions of the tumor.

## Conclusions

Herein we report the effectiveness of two Pcs to inactivate glioblastoma cells in culture. Both ZnPc and its derivative TAZnPc are incorporated into the cells and, in combination with light, are able to trigger cell death mainly by apoptosis. This cell death mechanism involves activation of caspase 3 and loss of mitochondrial morphology and functionality, as well as of lysosomal integrity. Early markers of apoptosis (externalization of phosphatidylserine) and fragmentation of DNA were also observed. The development of novel modified PSs for the treatment of brain tumors will be of help to control this devastating disease and to promote PDT as an adjuvant therapy for their treatment.

## Materials and Methods

### Photosensitizers

All starting materials were purchased from Sigma-Aldrich. They were used without further purification. Column chromatography was performed on silica gel (70–270 mesh ASTM). UV spectra and fluorescence spectra were recorded on a Shimadzu UV-1800 Agilent Cary Eclipse Fluorescence Spectrophotometer. Microwave monomode CEM-Discovery reactor was used in the synthesis.

Zn(II)phthalocyanine (ZnPc) was obtained from phthalonitrile in DMF (dimethylformamide) via microware assisted synthesis^58^ and compare with commercially sample (Aldrich).

Zn(II)tetranitrophthalocyanine (TNZnPc). A mixture of 4-nitrophthalonitrile (346 mg, 2 mmol) and zinc(II) acetate dihydrate (109 mg, 0.55 mmol) in 3 ml of DMF were irradiated in a microwave oven at 100 W and 150 °C for 120 min. Then, the reaction mixture was cooled to room temperature and precipitated with 50 ml of water. The green solid was separated by centrifugation and washed with water. Yield: 86%.

*Zn(II)tetraminephthalocyanine (TAZnPc)* was synthesized from TNZnPc as previously reported^59^.

The products characterization UV-visible, fluorescence and ^1^HNMR are in Supplementary Material.

### Photochemical studies

Quantum yields of singlet oxygen photogeneration were determined using the relative method with unsubstituted zinc phthalocyanine and photooxidation of DMA (9,10-*dimethylanthracene)*^60,61^. The kinetics of DMA photooxidation was studied by following the decrease of the absorbance (A) at λ_max_ = 378 nm. The rate constants (k_obs_) were obtained by a linear least-square fit of the semilogarithmic plot of Ln A_0_/A vs. time. Solutions of DMA (35 µM) and PS in DMF (sensitizer absorbance 0.2) were irradiated in 1 cm path length quartz cells (2 mL) with monochromatic light at λ_irr_ = 670 nm, from a LED NES110NR lamp (Red (625 nm) Lustrous-green Technology of Lightings). ZnPc (Φ_Δ_ = 0.56) was used as a reference in DMF^33^. All the experiments were performed at 25.0 ± 0.5 °C. The pooled standard deviation of the kinetic data, using different prepared samples, was less than 10%.

TAZnPc fluorescence quantum yield Φ_F_ was calculated by comparison of the area below the corrected emission spectrum with that of ZnPc as standard, λ_exc_ = 640 nm, Φ_F_^ZnPc^: 0,17 (in DMF)^32^. Spectra were recorded using 1 cm path length quartz cells at 25.0 ± 0.5 °C.

### Light source

Irradiation was performed using a 150 W/21 V quartz halogen lamp (see spectrum in Supplementary Fig. 3). The light was filtered through a 2.5 cm glass cuvette filled with water to absorb heat at 5 cm of lamp. The sample was at 15.0 cm of lamp. A wavelength range between 350 and 800 nm was selected by optical filters. The light fluence rate at the treatment site was 12.5 mW/cm^2^ and the light doses of 10 and 27 J/cm^2^ (See Supplementary Material).

### Cell culture conditions

T98G, MO59, LN229 and U87-MG glioma cell lines (ATCC American Type Culture Collection) were cultured in DMEM (Dulbecco’s modified Eagle medium) supplemented with 10% of fetal bovine serum (FBS) and penicillin/streptomycin as antibiotic (Gibco) at 37 °C in a humidified incubator with 5% carbon dioxide atmosphere.

### Cell viability determination

Cell viability was determined using alamarBlue Cell viability reagent (Invitrogen) following the protocol provided by the manufacturer. Briefly, cells were incubated with the reagent alamarBlue (10 µL) dissolved in 90 µL of DMEM supplemented with 10% of FBS (final volume: 100 µL in 96 multiwell plate), during 4 hours at 37 °C. Fluorescence measurements were done using a Biotek microplate reader with an excitation wavelength of 540–570 nm and the fluorescence emission reads at 580–610 nm.

### Subcellular localization of PSs

T98G were seeded onto coverslips and cultured overnight as described at cell culture conditions. Pc at final concentration of 0.5 μM were added diluted in DMEM supplemented with 4% of FBS and antibiotics, and incubated during 18 hours. Then, the medium was discarded, the cells washed twice using phosphate buffer saline (PBS) 1X and incubated with organelles specific probes: LysoTracker Green (lysosomes) and MitoTracker Green (mitochondria) following the manufacturer instructions (Invitrogen). After incubation, medium with probes was discarded, freshly medium added to cells and then the cells were observed *in vivo* under confocal microscopy. The sensitizer fluorescence was monitored with an Olympus FV1200 confocal microscope using the following settings: Excitation at 635 nm and emission evaluated at 660–720 nm for ZnPc and 700–730 nm for TAZnPc. The organelle specific probe fluorescence was monitored using the settings recommended by the manufacturer. Image analysis and the Pearson’s correlation coefficient calculated was performed using ImageJ software.

### Cellular uptake

Cellular uptake was determined by two methods, direct measuring of fluorescence^62^ and flow cytometry^63,64^. T98G cells were seeded in a 24-well plate (75000 cells/well) and incubated at 37 °C for 24 hours. The different Pcs were added to cells at concentration of 0.5 µM and incubated during 1, 3 and 18 hours in absence of light. Medium was removed, cells washed twice with 500 µL of PBS, counted and lysed using 500 µL of SDS 2% during 1 h. The concentration of Pc in the cell lysate was determined by dilution of 300 µL of cell lysate with 200 µL of DMF and measuring the fluorescence of the samples (ZnPc: λex:640 nm, λem:675 nm, TAZnPc λex:670 nm, λem:711 nm) using an Agilent Cary Eclipse Fluoresence Spectrophotometer. Calibration curves were prepared by dilution of Pc stock solution in DMF at desired concentration in cell lysate (300 µL) diluted in DMF (200 µL). For flow cytometry, cells were trypsinized, centrifuged and resuspended in PBS 1X. Pc fluorescence was determined using the following cytometer settings: excitation at 633 nm and emission acquired using a long pass filter (780 ± 60 nm) in a Becton Dickinson FACSCanto II cytometer.

### Dark cytotoxicity

To evaluate the cytotoxicity of Pc in the absence of irradiation, T98G cells were seeded in 96 well plates (7000 cells/well) and incubated for 24 hours in DMEM supplemented with 10% of FBS plus antibiotics. Then, medium was replaced and the cells were incubated in the presence of different concentration of the PS dissolved in DMEM supplemented with 4% of FBS plus antibiotics. After 18 hours of incubation, medium was discarded and viability was determined using alamarBlue (Invitrogen) as described above.

### Photocytotoxicity

Cells were seeded in 96 well plates at a density of 7000 cells/well and grown overnight at 37° in DMEM supplemented with 10% of FBS plus antibiotics. Medium was replaced with DMEM supplemented with 4% of FBS containing the photosensitizer at the desired concentration and cells were incubated for 18 hours at 37 °C. Then, the culture medium was replaced with 10% FBS supplemented DMEM medium and cells were irradiated using a homemade device at the desired light dose (10 or 27 J/cm^2^). Light dose was determined using a SE-9087 Digital Light Meter (Extech Instruments). Viability was examined 24 hours after illumination using alamarBlue (Invitrogen) as described previously and expressed referred to control cells (cells non-irradiated without Pc).

### Clonogenic Assay

Cells were treated as described above in Photocytotoxicity and 24 hours after, cells were trypsinized, counted, diluted and plated. Plates were returned to the incubator for 8–10 additional days. Then, cell media was removed and cells rinsed using 1X PBS and stained using 500 µL of fixing/staining solution (0.05% crystal violet, 1% formaldehyde, 1% methanol in PBS 1X) during 30 minutes at room temperature and washed using water. After air drying, plates were photographed and counted. Survival fraction was calculated as (number of colonies formed/number of cells seeded × plate efficiency) × 100. Plate efficiency was calculated using control cells and the following formula: PE = (number of colonies formed/number of cells seeded). All experiments were carried out in triplicate and data presented as mean values ± SEM.

### Immunodetection of cleaved caspase 3

Cells were seeded on slides in 24 multiwell plates at a density of 150000 cells/well and grown overnight at 37 °C. PDT treatment was performed as described above; then, cells were washed twice with cold PBS 1X and fixed using 4% paraformaldehyde at room temperature for 10 min. Fixed cells were permeabilized in PBS 1X Triton X-100 0.1% during 10 min at room temperature and then washed twice using PBS 1X. Coverslips were blocked with PBS 1X/Bovine Serum Albumin (BSA) 3% during 2 hours at room temperature and incubated overnight at 4 °C with anti-cleaved caspase 3 (1:400, Cell Signaling). After washing, cells were incubated with anti-rabbit Alexa 546 antibody (1:1000, Molecular Probes, USA) for 1 hour at room temperature. Nuclei staining was done during 10 min at room temperature by incubation with 4’,6-diamidino-2-phenylindole (DAPI). Coverslips were mounted with FluorSave (Calbiochem, San Diego, USA) and visualized under an Olympus FV1000 confocal microscope. For the quantification, at least 10 microphotographs of cells submitted to each treatment were analyzed.

### Detection of apoptosis and necrosis: Annexin V – PI staining

The determination of cell death mode was performed three hours after PDT. Floating cells were collected and washed with PBS 1X. Attached cells were collected by incubation with trypsin during 2 min. Cell surface exposed phosphatidylserine was evaluated using Annexin-V FITC conjugate (Invitrogen) following the manufacturer´s recommended protocol. Cells were incubated also with PI and then analyzed by flow cytometry. Cells were classified as follow: alive cells (Annexin V−PI−), cells undergoing early apoptosis (Annexin V+ PI−), cells undergoing late apoptosis (Annexin V+ PI+) or cells undergoing necrosis (Annexin V− PI+).

### Mitochondrial membrane potential (MMP)

To evaluate the mitochondrial membrane potential after PDT, cells were stained using MitoTracker red CMXRos and Annexin-V FITC conjugated (both from Invitrogen). Three hours after PDT, cells were collected by trypsinization and stained using MitoTracker red CMXRos for 30 min as recommended by the manufacturer. After washing, cells were incubated with Annexin V FITC conjugated and analyzed by flow cytometry. The mitochondrial morphology after PDT was assessed using MitoTracker red CMXRos staining as described above. After staining, cells were fixed, the coverslips were mounted with FluorSave (Calbiochem, San Diego, USA), visualized under an Olympus FV1000 confocal microscope and the images were acquired with the XYZ resolution needed for restoration by deconvolution. The stack of images was deconvolved using Huygens Deconvolution software (SVI, Netherlands).

### Lysosome disruption after PDT. LysoTracker Green retention assay

In order to evaluate the integrity of lysosomes after PDT we analyzed the capacity of the cells to retain the organelle specific probe LysoTracker Green after PDT following the protocol described previously^65^. Briefly, cells were incubated with LysoTracker Green at concentration of 25 nM dissolved in DMEM supplemented with 10% FBS and antibiotics during 30 minutes. After this, medium was discarded and new fresly medium was added to ceels prior irradiation. One hour after irradiation cells were collected and analyzed by flow cytometry. Mean fluorescence intensity was obtained and compared to cells without irradiation.

### DNA fragmentation analysis

DNA fragmentation analysis was performed as described previously^66^. Briefly, after PDT treatment (6 or 24 hours), adherent and non-adherent cells were collected by centrifugation at 1200 rpm during 5 min and washed using cold PBS 1X. Then, cells were lysed in ice-cold lysis buffer (0.15 M NaCl, 10 mM Tris-HCl pH 7.8, 2 mM MgCl_2_, 1 mM DTT and 0.5% NP-40) during 40 min on ice. Lysates were centrifuged at 1500 rpm during 10 min, pellets were resuspended in ice-cold buffer containing 0.35 M NaCl, 10 mM Tris-HCl, 1 mM MgCl_2_ and 1 mM DTT and incubated on ice for 20 min. Then, DNA was extracted with phenol-chloroform and precipitated with 0.01 M MgCl_2_ and 2.5 volumes of 100% ethanol overnight at −20 °C. DNA was collected by centrifugation at 14000 rpm for 20 min, resuspended in 10 mM Tris-1 mM EDTA with 0.1 mg/mL RNase A, and incubated at 37 °C for 1 hour. Finally, DNA electrophoresis was performed in 1.5% agarose gel containing ethidium bromide and visualized under UV light.

## Supplementary information

Supplementary material
