# Supplementary material for: Effectiveness of ZnPc and of an amine derivative to inactivate Glioblastoma cells by Photodynamic Therapy: an in vitro comparative study
Source: Sci Rep. 2019 Feb 28;9:3010. doi: 10.1038/s41598-019-39390-0 (PMC6395748; doi:10.1038/s41598-019-39390-0)
Supplement: Supplementary file 1 — Supplementary material [file 41598_2019_39390_MOESM1_ESM.pdf]

# Effectiveness of ZnPc and of an amine derivative to inactivate Glioblastoma cells by Photodynamic Therapy: an *in vitro* comparative study.

*Fabiola N. Velazquez*[1], *Mariana Miretti*[2], *Maria T. Baumgartner*[2], *Beatriz L. Caputto*[1], *Tomas C. Tempesti*[2,*] *and César G. Prucca*[1,*]

[1]CIQUIBIC (CONICET), Departamento de Química Biológica, Facultad de Ciencias Químicas, Universidad Nacional de Córdoba, Córdoba, Argentina

[2]INFIQC (CONICET), Departamento de Química Orgánica, Facultad de Ciencias Químicas, Universidad Nacional de Córdoba, Córdoba, Argentina

[*] Corresponding authors Cesar G. Prucca. E-mail: cprucca@fcq.unc.edu.ar and Tomas C. Tempesti, E-mail: tempesti@fcq.unc.edu.ar

## Supplementary Figures

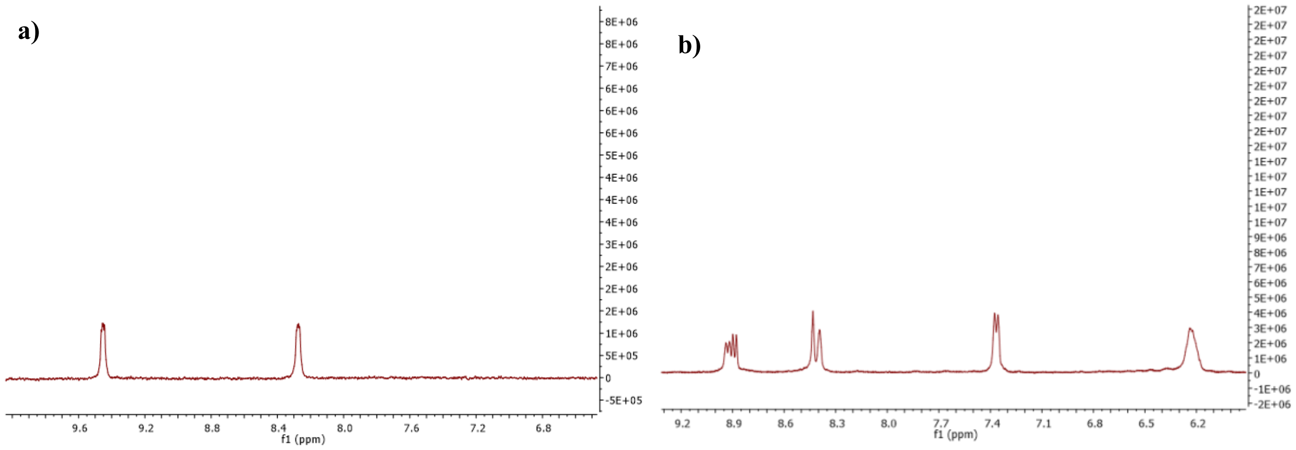


**Supplementary Figure 1.** *Spectroscopic data of synthetized Pcs*. HNMR profile of ZnPc(a) and TAZnPc (b) was determined using Bruker Advance-400 MHz corresponding both with those previously reported[1,2].

$^1$HNMR (DMSO $d_6$) a) ZnPc b) TAZnPc

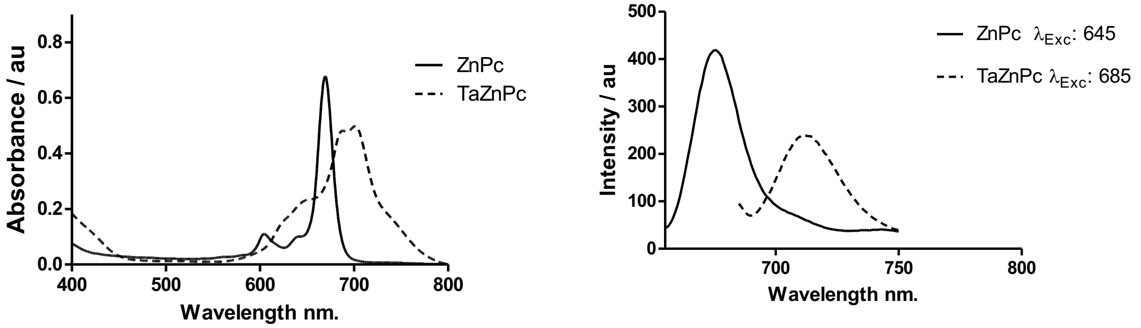


**Supplementary Figure 2.** *Pcs UV-vis and fluorescence spectra.* UV-vis (left) and fluorescence spectra (right) of ZnPc (continues line) and TAZnPc (dashed line) were recorded in dimethylformamide DMF. UV spectra and fluorescence spectra were recorded on a Shimadzu UV-1800 Agilent Cary Eclipse Fluorescence Spectrophotometer

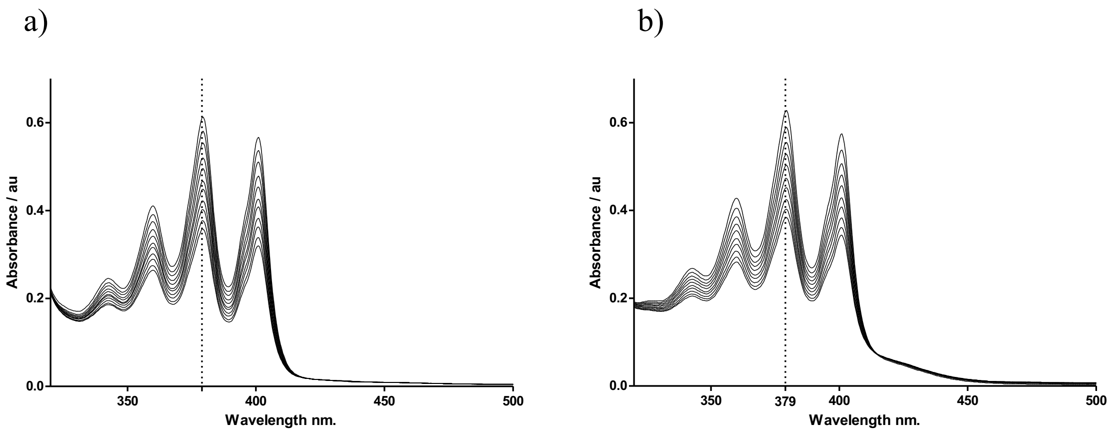


**Supplementary Figure 3.** *Photooxidation of DMA (9,10-dimethylanthracene) for the determination of TAZnPc produced singlet oxygen*. a) Absorbance spectra of photooxidation of DMA using ZnPc as standard. b) Absorbance spectra of photooxidation of DMA obtained for TAZnPc singlet oxygen determination as described in materials and methods.

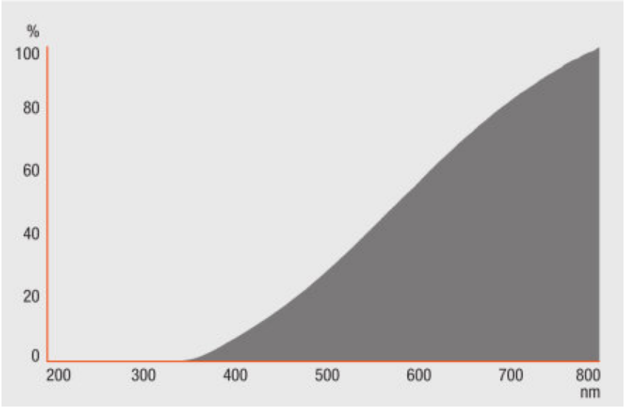


**Supplementary Figure 4**. *PDT light source Lamp Spectrum.* Lamp spectrum was obtained of lamp data (Osram).

*Light dose determination*

The light dose was determined weekly using an SE-9087 Digital Light Meter performing the measurements as an integration of all wavelength. We measured the intensity of the light source at the same distance from the cell cultures and during the same irradiation time. After the measurement was performed, the mean value of LUX obtained was multiplied by the device scale factor and converted to $w/m^2$ and after to $w/cm^2$. Irradiation times corresponding to 10 and 27 $J/cm^2$ were obtained weekly from the following formula:

Time (seconds)= [light dose ($J/cm^2$) x surface ($cm^2$)]/lamp power ($w/cm^2$)

In this way, we take into account the variations inherent to the use of the device.

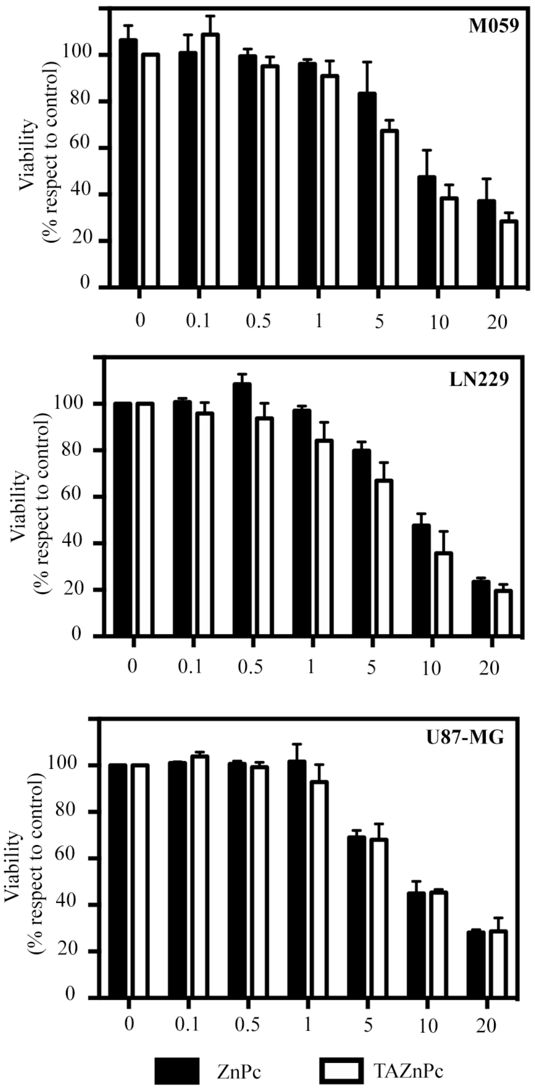


**Supplementary Figure 5.** *Dark toxicity of photosensitizers*. MO59 (upper row), LN229 (middle row) and U87-MG (lower row) cells were incubated in the dark during 18 hours with different concentrations of TAZnPc (white bars) or ZnPc (black bars) dissolved in DMEM supplemented with 4% of FBS plus antibiotics. Then the cells were washed and viability assessed using alamarBlue as described under Materials and Methods. Results are presented as mean percentage of viability with respect to the control (cells without Pc) ± SEM of three independent experiments made in triplicate.

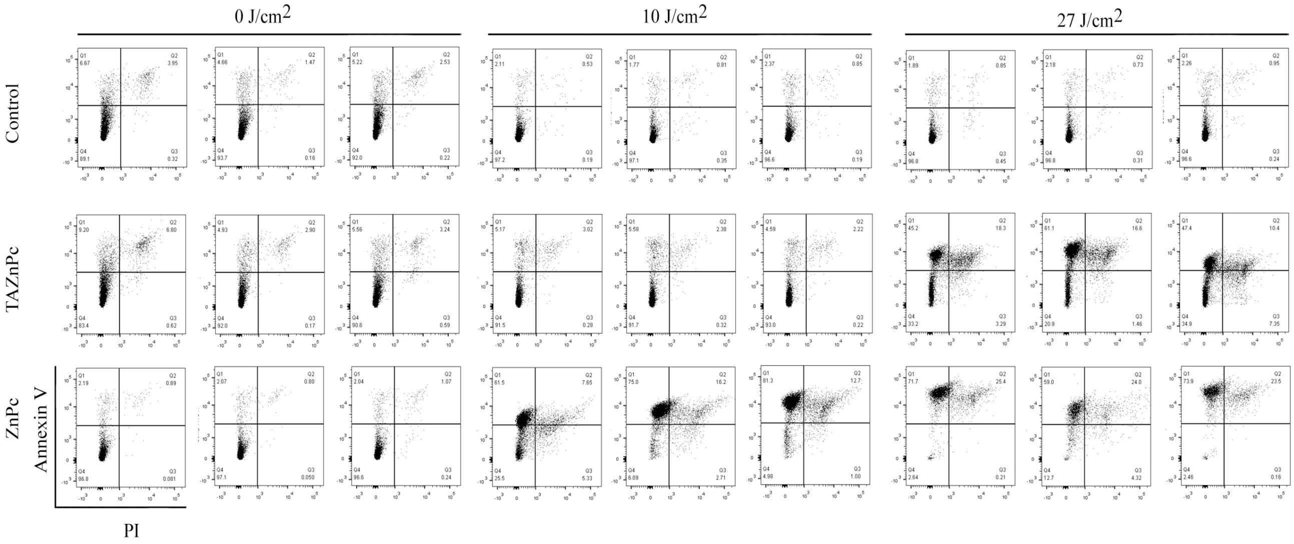


**Supplementary Figure 6.** *Apoptosis induction: Annexin V/PI staining.* T98G cells were incubated with TAZnPc or ZnPc dissolved in DMEM supplemented with 4% FBS plus antibiotics at a concentration of 0.5 μM during 18 hours. Then, medium was replaced by fresh medium not containing Pcs and cells were irradiated using two light doses: 10 J/cm$^2$ or 27 J/cm$^2$. Three hours after irradiation, floating and attached cells were collected and washed. Then cells were stained using Annexin V conjugated to FITC (staining used to determine exposed phosphatidylserine) and PI and analyzed by flow cytometry. Dot plot graphs (triplicates) form an experiment out of three is shown. Cells in the lower left quadrant are considered living cells (Annexin V - PI -), cells in the upper left quadrant are early apoptotic cells (Annexin V + PI -), cells in the upper right quadrant are late apoptotic cells (Annexin V + PI +) and cells in the lower right quadrant are necrotic cells (Annexin V- PI+)

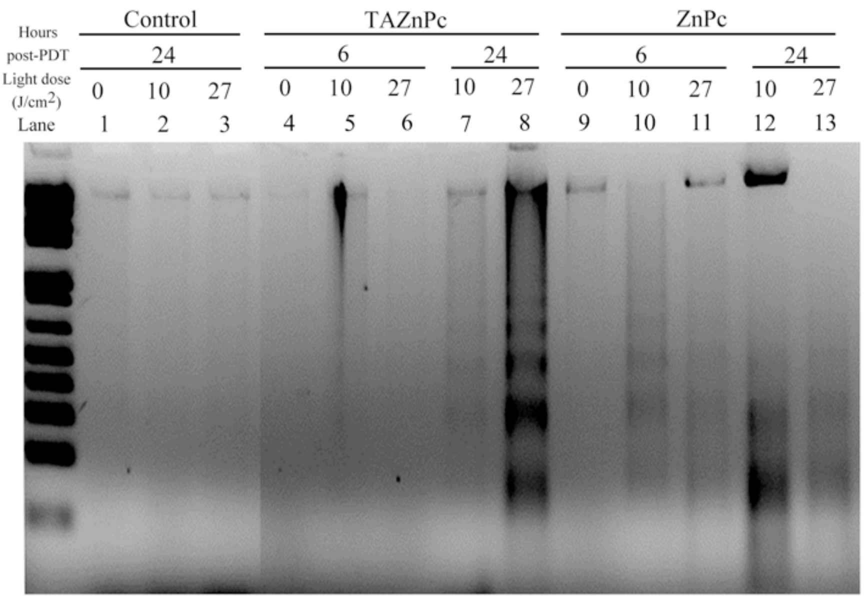


**Supplementary Figure 7.** *Apoptosis induction: DNA laddering*. Cells treated with the different Pcs were used to isolate total DNA and evaluate the genomic integrity after PDT. For this, 6 or 24 hours after PDT, cells (floating and attached) were collected and DNA isolated as described under Materials and Methods. DNA was separated by electrophoresis in agarose gel containing ethidium bromide. DNA laddering pattern, characteristic of apoptosis, is clearly visible 24 hours after PDT in cells treated with TAZnPc and irradiated with 27 J/cm$^2$ (lane 8) and 6 hours after PDT in cells treated with ZnPc and irradiated with 10 J/cm$^2$ as well as in those irradiated with 27 J/cm$^2$ (lane 10 and 11).

## References


1. Cong, F. D. *et al.* Facile synthesis, characterization and property comparisons of tetraaminometallophthylocyanines with and without intramolecular hydrogen bonds. *Dye. Pigment.* **66,** 149–154 (2005).
2. Conte, C. *et al.* Nanoassemblies based on non-ionic amphiphilic cyclodextrin hosting Zn(II)-phthalocyanine and docetaxel: Design, physicochemical properties and intracellular effects. *Colloids Surfaces B Biointerfaces* **146,** 590–597 (2016).
